# Developing an Innovative Medical Training Simulation Device for Peripheral Venous Access: A User-Centered Design Approach

**DOI:** 10.3390/healthcare8040420

**Published:** 2020-10-22

**Authors:** Constanza Miranda, Fernando Altermatt, Ignacio Villagrán, Julián Goñi

**Affiliations:** 1 DILAB School of Engineering, Pontificia Universidad Católica de Chile, Santiago 7820436, Chile; jvgoni@uc.cl; 2 Department of Anestesiology, School of Medicine, Pontificia Universidad Católica de Chile, Santiago 7820436, Chile; falterma@uc.cl; 3 Health Sciences Department, School of Medicine, Pontificia Universidad Católica de Chile, Santiago 7820436, Chile; invillagran@uc.cl

**Keywords:** design based research, academic use of simulation, health education, simulation technology, anthro-design, health innovation

## Abstract

Nurses and other health students may lack the proper time for training procedural tasks, such as peripheral venous access. There is a need to develop these abilities in novices so that errors can be avoided when treating real patients. Nonetheless, from an experiential point of view, the simulation devices offered in the market do not always make sense for educators and trainees. This could make the adoption of new technology difficult. The purpose of this case study is to describe the development of an innovative simulation device and to propose concrete tactics for the involvement of the educators and trainees. We used a participative design based approach, with an ethnographic basis, where incremental cycles of user testing, development and iteration were involved. The study showcases methods from the field of design and anthropology that can be used to develop future simulation devices that resonate with students and educators to achieve a long term learning experience. Results could shed a light on new ways for the involvement of educators and students to create devices that resonate with them, making learning significant and effective.

## 1. Introduction

In their formative process, health professionals acquire different procedural skills by level and discipline. One of the main issues in teaching procedural tasks is that instruction is based on the master-apprentice model [1] (Wigton, 1992. The master executes an action and the apprentice imitates the procedure under the informed and critical supervision of the trainer. This model is known as “see one, do one, teach one” [2,3]. 

There are multiple issues with this teaching model. First of all, there are ethical issues with exposing patients to procedures executed by individuals with no experience (novices) [4]. Second of all, there are limitations in the amount of procedures and attempts that the apprentice can execute under direct supervision due to the alternative cost of having an expert dedicated to that instance. Lastly, there are limitations in the quantity and quality of the educational feedback that the instructor can give to the trainee, making it hard to determine if a trainee has reached the learning objectives expected by a patient without direct supervision [5]. 

During the last 15 years, and to solve these issues, the use of simulation has been proposed as an alternative to improve the learning and teaching experience in health professionals [6,7]. Thus, multiple institutions have implemented successful training programs based on simulation [6,8]. A study published by the Journal of Nursing Regulation in 2014 provides substantial evidence that up to 50% of simulation can effectively substitute traditional clinical experience in all prelicensure core nursing courses [9]. 

One of the key procedural techniques for nursing students to learn during their undergraduate studies is how to install peripheral intravenous accesses [10]. Complications derived from an incorrect execution of this procedure in real patients include bleeding, extravasation, hematomas, or infections [11,12,13]. Even though there is consensus on the need for training and for the use of simulation as means for instruction, there are practical issues in the existing simulation devices currently available. As Carlson et al. indicate: “Few objective metrics exist to quantify differences between providers of various skill levels” (p. 1) [14].

Conceptually, the benefit of using an objective metrics device that is autonomous is twofold: it allows repetitions without the need for a physically present instructor, and it provides a reliable form of feedback that is independent from the number of attempts or students considered in the learning session. 

In order for the device to be usable and intuitive and to fulfill the expectations of learners and instructors, a user-centered design approach becomes key. User centered design involves widely User Experience (UX). Unlike market research, user research involves three main components: (1) meeting an important user need (“Usefulness”); (2) being usable (“Usability”); and (3) evoking positive emotions through look and feel (“Desirability”)” [15]. When a product is designed successfully with the different end-users in mind, the possibilities of embracing a new technology increase [16]. As Miranda asserts, the human interface must be accounted for when designing solutions for real people. Through the active involvement of the users, stakeholders, and key informants we are able to embrace the human complexities of our innovation challenges [17].

Considering that the costs in simulation are very variable and not always more affordable than traditional clinical placements [18], adoption of equipment becomes relevant. Literature states that resources available to provide education are finite; therefore, for the implementation of innovations in health education to be successful, they must be submitted to previous economic evaluations [19]. People-centered innovation shies away from a purely economic model [20] and looks to what makes the user experience memorable. This could be key to not only the adoption of a new device, but also to embodying immersive and sensory characteristics that are key to a significant educational experience.

A considerable body of academic work has been dedicated to studying the educational effect of simulation [21]. Nonetheless, most studies on simulation in nursing education have not adopted learning-centered research designs and explanations [22] or learning theory at large [23]. All complex and human psychological processes (higher psychological processes), such as learning itself are embedded into history and culture, but also structured through basic bodily processes (lower psychological processes). Learning does not occur in the brain alone, but all across the body and it involves the student as a whole. For simulation-based nursing education, this means that we have to consider all integrated dimensions of the conscious experience: the psychomotor, the cognitive, the emotional and all bodily systems in general. Additionally, learning is co-constructed by interaction with an “other”. This other is not necessarily a human person, but rather, something acting as a human person. Physical objects can also act as socializing agents for development under a socio constructivist framework [24]. Simulation devices operate as a collaborating other and thus devices should be designed to be interactive, emotionally supportive and feedback-giving (as collaborating with humans is). This sort of integral approach to learning becomes key when trying to change the perception of an uninvolved student to that of an active apprentice in control of their own training. A participatory approach could benefit students by making them emotional owners of their own learning process [25]. It could also benefit faculty staff by providing student feedback directly and engaging them as a partner in teaching and learning [26]. The main purpose of this study is to describe the process undertaken for the design and development of an intravenous peripheral access simulation device and shed a light on the participative design, anthro-design for health and educational principles of creating new technologies that resonate with the final users. In this article we aim to address the following research question: How are user-centered design tactics deployed when creating a new educational device that resonates with the educator and the trainee? What are the main strategies to follow? 

## 2. Materials and Methods

### Methodology: An Anthro-Design Process Involving the Users

The case portrayed in this article is a device developed by a team involving multidisciplinary academics and practitioners at Pontificia Universidad Católica de Chile. The research behind the process complied with all the Institutional Review Board’s (IRB) processes (e.g., informed consents). The case took place between the years 2014 and 2019 and it relied heavily on human/people centered design and user experience methodologies [27,28,29]. In particular, this research project used the anthro-design methodology as described by Miranda [17]. 

**Figure 1:**
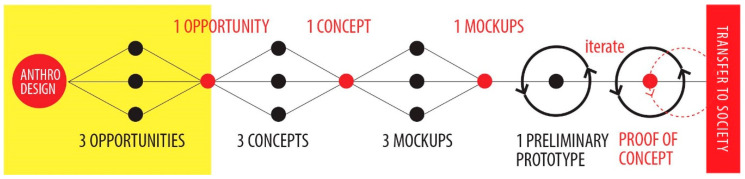
The Anthro-design process based on human oriented research (Miranda, 2019) [17].

We used anthro-design, a mix between the qualitative research methods used in anthropology and the visual and prototyping design techniques [17,30]. We also followed the traditional divergent and convergent design process [31,32]. This is summarized in Figure 1. The key to this strategy is that the team starts by understanding the cultural values underlying the communities they are working with (in this case, trainees and trainers in the nursing profession) to identify drivers, values and expectations related to the experience. It also involves the ethical considerations of a cultural anthropology framework. Data is collected in the field by using rigorous ethnographic research methods [33,34,35] to frame and tackle a large design space [36]. 

Having a large design space implies that there is room for innovation and experimentation. Nobody comes with a preconceived idea of what should be done. On the contrary, opportunities for innovation are found in gaps and impressions of everyone involved, achieving a product that is multivocal [37]. That is, a product that represents different voices. The qualitative data is collected through open-ended interviews [38,39,40], field participant and non-participant observation and other forms of ethnographic immersion [38,41,42]. Later on, the data raised in the form of fieldnotes, pictures and other audiovisual material is analyzed and coded using Qualitative Data Analysis (QDA) using Grounded Theory [43,44]. At the beginning, this process of constructing the research protocols (i.e., for the interviews) is very exploratory in nature, but as the process advances, questions and analysis become more and more structured.

After some of the fieldwork is carried out and data is analyzed, the team gathers to converge and detect opportunities or possible directions to follow. Then, in the form of a discussion that considers every point of view raised during the fieldwork, the team converges on one opportunity that should be pursued (usually is the most pertinent one to the context). Then, three concepts or possible ideas are developed by the team with the use of the Brainstorming technique [17]. After the team settles on one idea or concept, several prototypes are constructed and presented to the trainers and trainees for feedback and iteration in short and cheap development cycles [45]. All the team and diverse experts were involved in these stages. The peripheral venous access simulation device was designed translating the requirements, emotional drivers, cultural values, and expectations of the students and educators. The team had to translate that information into design attributes to be incorporated in the final model. 

**Figure 2:**
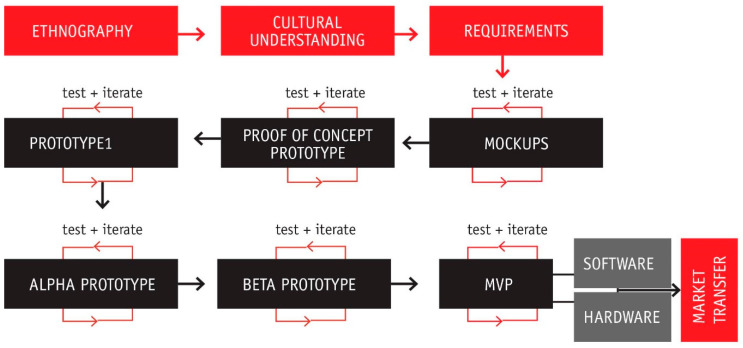
A general look at the process going from ethnographic data to the development of an MVP.

Figure 2 shows the major steps in the consecutive process of testing and developing the different prototypes for this particular device. All of these stages will be explored, in detail, in the results section.

**Figure 3:**
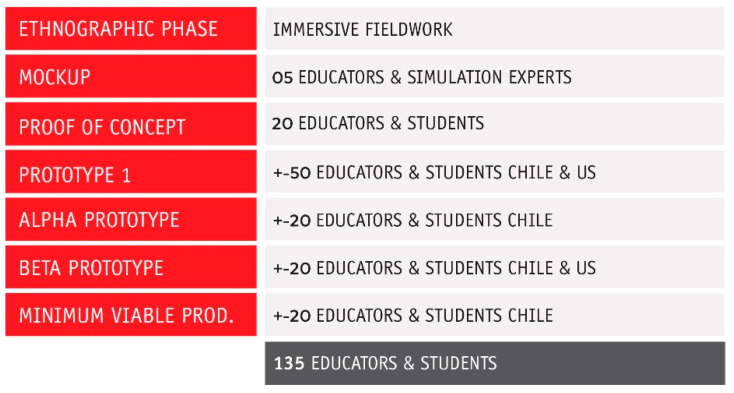
Summary of participants in the testing phases.

The team faced the different stages of the prototype with at least 135 educators and students in Chile and the United States (Figure 3). Being a qualitative study in nature, the number of participants was not as important as the depth in the rounds of testing and the iterations with the prototypes. Fortunately, we were able to expose some of the prototypes and functionalities to educators in Chile and the United States (East and West coast). 

## 3. Results

### 3.1. Minimum Viable Product and User Centered Design Process

Acknowledging the benefits of simulation for teaching procedural tasks and the lack of objective metrics in the assessment of learning, a multidisciplinary team belonging to the medical and engineering-design areas decided to develop a peripheral venous access simulator. The final version portrayed in Figure 2, shows the final minimum viable product (MVP) attained. 

**Figure 4:**
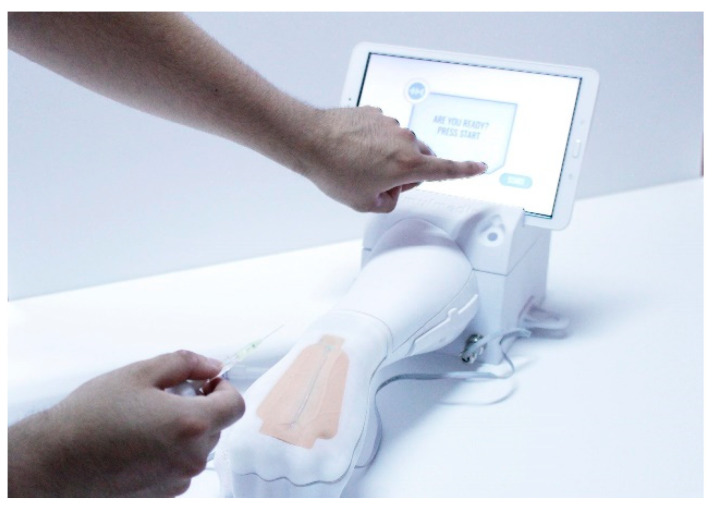
Minimum viable product developed by the team (2019).

**Figure 5:**
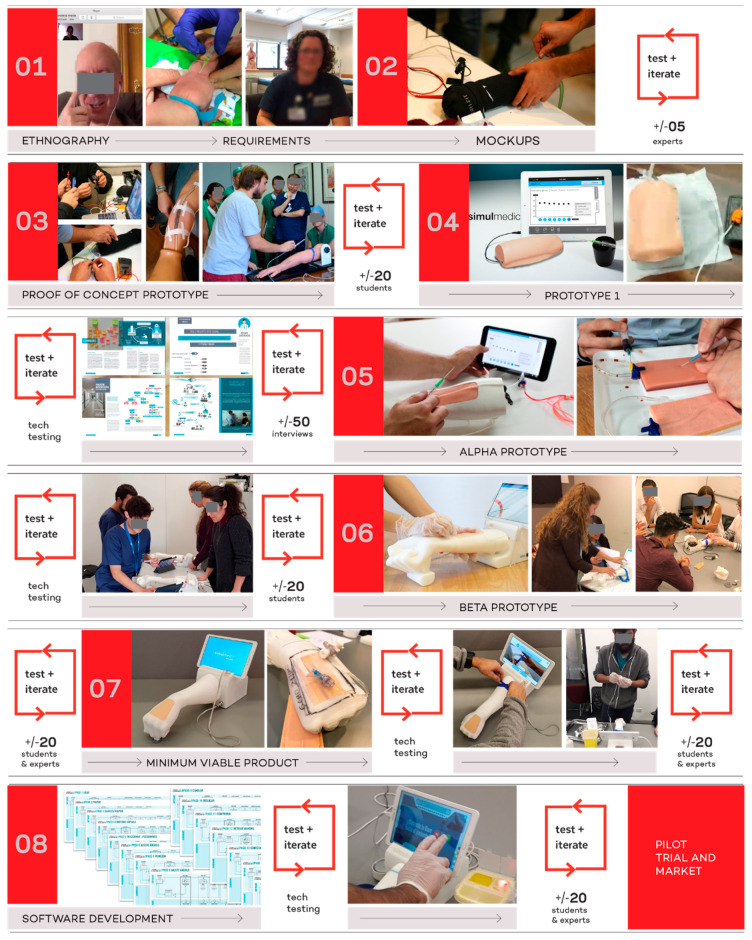
Process for development of the Peripheral Access Simulator between 2014 and 2019.

The final device, as seen in Figure 4, comprises hardware and software for autonomous training in peripheral venous access. The hardware involves a phantom arm, an instrumented needle and a software where the trainee interacts using a touchscreen. This minimum viable product (MVP) is absolutely functional and looks to fit the interests of trainers, trainees and educational institutions. Figure 5 portrays the major milestones in the overall design process to develop the MVP. The evolution of the device comprises at least 6 different typologies of hardware prototypes, from low fidelity to high fidelity, and one major stage related only to the development of software. The software entails the development of the User Interface (UI) and the User Experience (UX). Every stage includes a technology driven test phase, a test of the interaction with students and a testing phase with instructors or experts. This leads to fast iteration of the device and to the incorporation of the diverse users’ points of view and expectations throughout the design process. 

### 3.2. First Design Cycle: From Behaviors to Requirements, to Functional Prototypes

#### 3.2.1. Mockups: Going from Behaviors to Requirements in Cheap Physical Prototypes

The development process first started with an immersion in the nursing education field by a team of engineering-designers. The applied ethnographic field work consisted in visiting several training institutions, universities and hospitals to understand the cultural values underlying the educational practice in simulation and procedural learning in peripheral venous access. These field data are transcribed and analyzed using Grounded Theory [43]. Themes were created inductively by identifying patterns and similarities across different open codes that emerged from the interviews [43]. This immersion was key to identify gaps in the existing devices that could lead to innovation. As we stated before, there are practical issues in the existing simulation devices currently available on the market. What the team identified during the first immersion stage are the following concerns about current devices: (1) they are an expensive resource due to the alternative cost derived from the participation of a qualified instructor who provides feedback; (2) they are hard to scale, meaning that not all of the students get to puncture or interact with the device under direct supervision more than once; and (3) there is a difficulty to transfer subjective observations into meaningful feedback. After this, the team created a table that summarized the emotional and physical requirements that a new design should involve. After ideation, sketching and defining the pros and cons of the ideas, one concept was selected. In December of 2013, the first functional mockup (rapid and cheap prototype) was constructed. This iterating mockup was useful to test with trainers and trainees and to better understand what their requirements and expectations were. 

#### 3.2.2. Consecutive Prototypes and User-Based Iterations 

Diverse testing situations were put in place to see if the mockups and their basic functionalities fulfilled the requirements of the users. The iterations lead to the development of the first proof of concept prototype (a prototype that is not finished, it is an “unpackaged” technology, but it achieves the purpose of showing the functionalities that the product should bring to the users). 

### 3.3. Second Development Stage: Prototype Iterations with the Users

In these stages, feedback was not only focused on task-oriented testing (complete a task and evaluate it). Semi-structured and open ended interviews [38,39,40] were carried out and they were key to uncovering issues related to current simulation devices and they gave the possibility for the participants to give us ideas about better ways to improve a future device. Some examples can be illustrated in the following comments with participants:

This is an excerpt from an open-ended interview with a group of nursing educators in California:

*“We have three IV trainers that serve 100 students, we understand the gap. There is a need for this to be scalable. We need it to be used in multiple sites for multiple students. We have 5 campuses for nurse programs.”*

(Director Health Science Simulation Center at the Nursing School).

Our team translated this information into the following requirements: scalability of the device (that more people could train on it) and that more of them could be acquired by the institution to be placed in different sites. In another interview, the participants indicated:

*“You are breaking the barrier between the user and the product!”*

(Simulation Specialist at the Health Science Simulation Center at the Nursing School)

*“My job is to get the technology out of the way of the educators”. “There are so many different levels: equipment and the actual operations. The SIM technology is not very well designed, it is not very fancy technology. I want the technology to be invisible, so reliable that there isn’t that uncertainty if it will work or not. In my ideal world, there isn’t a need for an operator.”*

(Simulation Technician, Nursing School)

For some of the instructors and technicians, the experience with actual simulators was not really taking into account the needs of the educators. An important amount of the interviewees complained that the institution usually had to hire someone to fix or adapt the simulators, since they were not technologically robust and that using the service warranty would just interrupt the use of a simulator for some time. Our team translated this information into a requirement for creating a robust and intuitive technology that allows the simulator to be more autonomous and reliable. This is why the front-end of the software (UX/UI) aims to be graphically and interactively natural to any novice user. 

**Figure 6:**
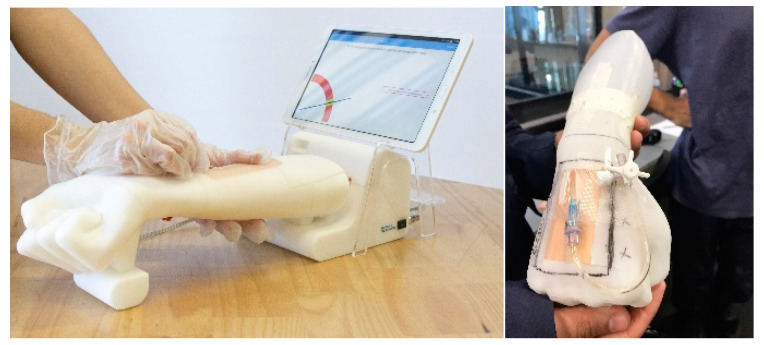
The prototype changes as the feedback from users is incorporated. In this case it involves switching the patch and the position of the hand.

Other particular iterations considered comments like: the idea of using a blood pump to have a realistic flow of the liquids or changing the position of the puncture patch as we see in Figure 6. 

## 4. Discussion

This case study is a contribution to research for the development of partial task simulators for nursing education. The results of our development process could shed a light on new ways to involve educators and students to create devices that resonate with them making learning significant and effective. During our fieldwork done in California, Boston, and Santiago de Chile, educators and students would complain about the lack of robustness of existing technologies in simulation. Furthermore, they perceived that simulators currently lack a satisfying user-experience that considers the point of view of the educator and the trainee. While doing the benchmark, we realized that simulation devices in this area had not been very innovative in incorporating technologies that could improve these gaps in the experience (UX). Through the use of an anthro-design methodology, we were able conduct a research and development (R&D) process to create an alternative device that we perceive to be more founded on the needs, habits and motivations of students and teachers. The device delivers successfully value to the users by being intuitive, matching their expectations on the experience (resonates with their expectations), and allowing autonomous peripheral venous access training (without the presence of an on-site instructor). 

In the first phase of the process, the design principles were derived from theoretical knowledge and discussion with experts. Some preconceptions about the way students learn were challenged, and some of the original design principles were reinforced and validated, others were discarded. The empirical knowledge gained from one cycle was immediately implemented in the next one. This iterative and reflexive attitude is crucial in order to integrate such different sources of knowledge (educational theory, medical expertise, teachers’ practical knowledge, and students’ subjective experience). Ultimately, a great number of participants (about 135) and in depth data were needed to produce a final prototype that reflected all of the requirements identified in the research process. From the incorporation of a blood pump to the design of the interface, almost every major design decision was motivated by users’ participation. Overall, these results add to the growing interest in nurse education to adopt a more learning-centered approach [22]. Additionally, these results also add to the recent interest in nurse education to generate design principles to guide the development of simulation-based educational strategies [46]. In the following section, we will systematize the design tactics we inductively learnt during this research and development process.

### Design Tactics for Future Developments That Resonate with Educators and Students

**Figure 7:**
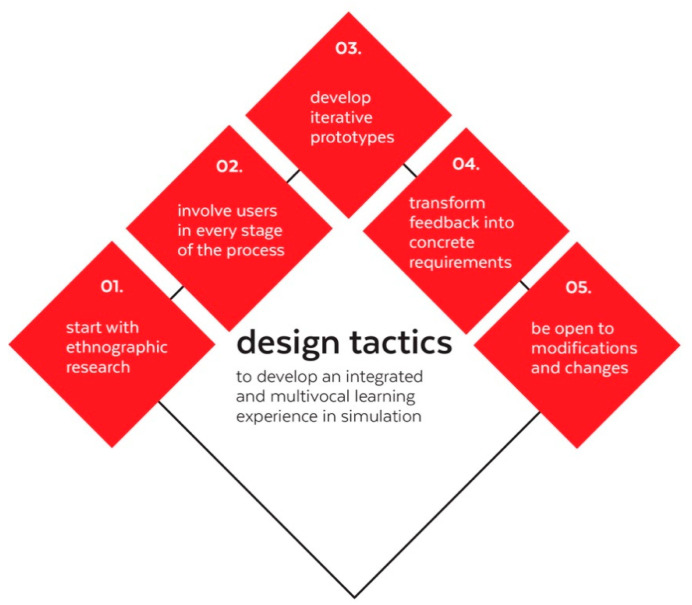
Design tactics used to design a multivocal learning experience.

The proposed methodology facilitated insight into the design process behind the development of a simulator that incorporates the point of view of educators and students to achieve multivocality [37]. Figure 7 exemplifies some of the important user centered design tactics that we identified throughout this study in order to achieve a simulation device that resonates with the end users. It is relevant to notice that these can be put in practice in the development of any simulation device that implies the use of hardware and software. Considering that, in simulation, learning is co-constructed by the interaction with the device, the focus is to achieve a successful interactive experience between a simulator and the individuals. Through these design tactics, we propose that other research and development processes can increase their odds in achieving that goal. The design tactics identified are the following:

1. Start with ethnographic research: Start talking to your users in their field: Anthro Design works wonders because it involves going in the field and understanding their day-to-day activities. In turn, this means that future simulation designers and practitioners would benefit from learning the basics of qualitative research and some of the fundamental tools (and theoretical foundations of those tools), such as observation, semi structured interviews and field notes.
2. Involve users in every stage of the process: Co-creating instead of convincing. Involve the user from the beginning and empower them to tell you about their ideas. People want to be heard. Additionally, we found that “snowball sampling” is key to testing with diverse institutions and individuals. When your participants are your allies, they can lead you to more participants.
3. Develop iterative prototypes: Start with cheap prototypes like mockups before investing in packaging a full technology. Prototyping several times to receive continuous feedback can be cheaper than developing and getting to the users with something that does not resonate with them. Forecast and plan for incremental development cycles and not just one field research and not just one development stage. It may seem expensive and time consuming to address it that way, but it is much cheaper than producing a final product that will not appeal to user or will not have a meaningful impact
4. Transform feedback into concrete requirements: Exercise translating qualitative information into design requirements. Sometimes it will not be too obvious, mostly because users do not think in terms of requirements (they think in terms of experience). However, creating requirements from data is a skill that can be developed over time.
5. Be open to modifications and changes: Do not impose your solutions. Perhaps one of the more challenging aspects of adopting an ethnographic approach is to suspend your previous judgments when entering the field. Under an anthro-design methodology it is equally important not to grow infatuated with your current ideas. Be open to learning and making mistakes. Do not start the process with a solution and do not rush to one once you get a little data. Trust the process and maintain an open mind.

Ultimately, all of these tactics point to a single strategy: Be people-driven, not only technology-driven. Devices should follow users’ guidelines, not the other way around. This also means that both “rational” (cold aspects of cognition), as well as “emotional/motivational” (hot aspects of cognition), should be considered. Simulation devices are not bound to the ideal learning scenario, but rather, to the human and complex scenarios of real-life teaching and learning. 

## 5. Conclusions

The contribution of this article is twofold. First, it presents the development of a partial task simulator that allows the autonomous training of students in peripheral venous access procedures. Second, it explores several learnings that arose from involving people in the actual design process to achieve a device that resonates with them. We have called these learnings “design tactics”. We conclude that adopting these design tactics will help other health simulation developers to bridge the gaps between technology and education by incorporating the requirements and expectations of their stakeholders in a systematic way. Finally, we believe this article will help anyone to improve the odds of a correct transfer and adoption of technology developed in academic environments to society. 

## 6. Patents

Altermatt, Fernando, Constanza Miranda, Benjamín Garnham, and Sanz-Guerrero Jorge. Medical Simulator for the Simulation of Puncture Operations. WO/2017/113022, issued 2017.

Altermatt, Fernando, Constanza Miranda, Benjamín Garnham, and Sanz-Guerrero Jorge. A medical simulator for the procedures associated with punctures for the simulation and training of interventions and punctures. It involves a phantom, an intervention device and a system for processing the communication and data between the phantom, the sensors, and detection means for the detection related to a group of membrane simulation and a base (translated). INAPI 59089. Granted in April 2020.
